# Supplementary material for: Earthquake lubrication and healing explained by amorphous nanosilica
Source: Nat Commun. 2019 Jan 18;10:320. doi: 10.1038/s41467-018-08238-y (PMC6338773; doi:10.1038/s41467-018-08238-y)
Supplement: Supplementary file 1 — Supplementary Information [file 41467_2018_8238_MOESM1_ESM.pdf]

Supplementary Information for: Earthquake lubrication and healing explained by amorphous nanosilica

Rowe et al.

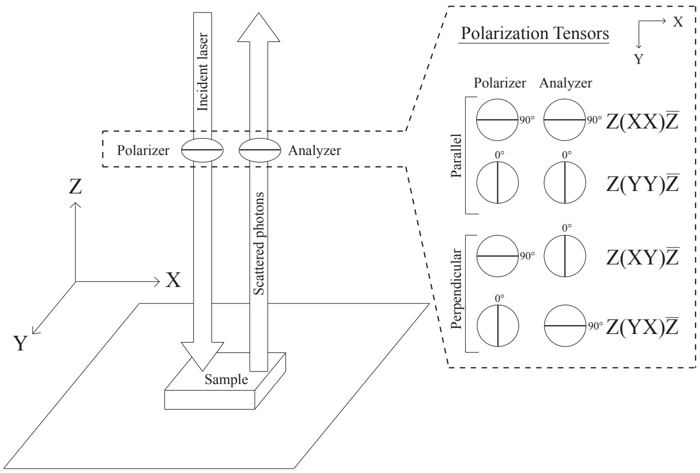

Supplementary Figure 1: Schematic showing orientation nomenclature for Raman polarization tensors

Supplementary Discussion

Our observations of the wear material show the polarization response which is widely observed for amorphous silica in this region (1; 2). The classic 4-tetrahedral D1 and 3-tetrahedral D2 ring modes often observed in vitreous silica are absent (or obscured) in the wear material (red bands in Figure 4A). The broad peak extends from the low wavenumber boson region (not shown) into the bending region of the so-called R band (δ_{bend} SiOSi). The broadening may be caused by a superposition of bands from large n-member rings of [$SiO_{4/2}$] tetrahedral (3) and Raman transitions due to a distribution in Si-O-Si bond angles (4). For more information on the formation of oligomeric rings in silica, the reader is directed to Trinh, et al. (5).

Though the absence of sharp peaks in the Raman spectrum of the wear material suggests that the quartz was converted to a vitreous silica, the spectrum is unlike previously published Raman spectra of glassy silica (6). The wear material was placed on a silicon wafer substrate in order to detect if the laser was interacting with the substrate; the signal from the silicon wafer would appear as a strong, narrow band at 520 cm^{-1}. This band was not observed, indicating that the spectrum is from the wear material alone. This is perhaps not surprising, given the different mechanism of formation of the nanopowder from quartz. It is known that the region between 10-1000 cm^{-1} can show considerable variation in band profile depending on density (3; 7)) and particle size (4). Bands due to silanol (Si-OH) and adsorbed water were too weak to be observed by Raman. Thus, the nanosilica wear material formed by shearing of quartz-rich rocks may have a distinctive Raman fingerprint, distinguishable from amorphous silica from other sources.

It was observed that the samples show fluorescence. In order to counteract this, points were irradiated with the laser for several minutes prior to data collection to photobleach the areas. The cause of the fluorescence has not been determined, but in principle, it could be caused by either metal impurities or the presence of organic material. Neither of these was identified in either EDS analyses (in electron microprobe and in transmission electron microscopy). As no impurity has been identified, the possibility exists that some characteristic of the amorphous silica causes the fluorescence.

Supplementary Note 1

Infrared (IR) spectroscopy measures a samples absorption of IR radiation over a range of wavelengths, using the principle that molecules will absorb light at frequencies that correspond with their own vibrational frequency.

The wear material has features in common with both the crystalline novaculite spectrum and the control nanosilica spectrum. Peaks and their positions in the different materials are compared to each other and to data from the literature in Table 2. The wear material has a weak shoulder at 1170 cm^{-1} and lacks a double peak in the 1050 cm^{-1} range, like the control nanosilica. However, the wear material is missing a peak at 967 cm^{-1}, which corresponds to the bending vibration of Si-OH, and it has a doublet at ∼780 and 800 cm^{-1}. Most telling, though, is the presence of a very small peak at 694 cm^{-1} in the wear material.

Infrared spectroscopy provides complimentary information. The wear material exhibits vibrational band patterns distinct from both the powdered novaculite starting material and a commercial sample of amorphous nano silica (Fig. 4B). The novaculite powder shows evidence of symmetric and asymmetric bending associated with Si-O-Si modes, typical of quartz (8). The wear material spectra display features which are absent in the novaculite, including absorbances for H_2O and silanol bending. The absorbances for stressed Si-O-Si bonds are locally more pronounced in the wear material (1086, 1043 cm^{-1}) or have different relative magnitudes (795, 775 cm^{-1}) than in the powdered Novaculite. The ratio of these peaks is lower at higher crystallinity (9). Ratios of 2.2 for the Novaculite powder and 6.0 for the wear material show the significant reduction in crystallinity during frictional wear. The wear material is also distinct from commercial nanosilica, which lacks some absorbance peaks indicative of stressed Si-O-Si bonds (1163 and 694 cm^{-1}).

The peak at ∼694 cm^{-1}, caused by the symmetric bending of Si-O, is known to only appear in crystalline phases (8; 9). The ratio between the ∼695 cm^{-1} and the ∼778 cm^{-1} peak has been used as a measure of crystallinity of silica based rocks (the 695 cm^{-1} is equivalent to our 694 cm^{-1} peak; 9). The peak at 695 cm^{-1} exists because of octahedral site symmetry, whereas the peak at 778 cm^{-1} is from the symmetric Si-O-Si stretching mode corresponding to tetrahedral site symmetry (9). Octahedral site symmetry is weaker and will be destroyed before tetrahedral site symmetry during structural damage, which allows for an estimation of the crystallinity of the silica by comparing the relative intensities of the two vibrational modes. A calculation of the ratio of the intensity of the 776 cm^{-1} peak and the 694 cm^{-1} peak of the powdered novaculite is 6.0, and of the wear material is 1.9, indicating a large decrease in crystallinity during production of the wear material.

Supplementary References

[1] Hehlen, B. & Simon, G. The vibrations of vitreous silica observed in hyper-raman scattering. *Journal of Raman Spectroscopy* **43**, 1941–1950 (2012).

[2] Rahmani, A., Benoit, M. & Benoit, C. Signature of small rings in the raman spectra of normal and compressed amorphous silica: A combined classical and ab initio study. *Physical Review B* **68**, 184202 (2003).

[3] Borowicz, P. *et al.* Deep-ultraviolet raman investigation of silicon oxide: thin film on silicon substrate versus bulk material. *Advances in Natural Sciences: Nanoscience and Nanotechnology* **3**, 045003 (2012).

[4] Alessi, A., Agnello, S., Buscarino, G. & Gelardi, F. Structural properties of core and surface of silica nanoparticles investigated by raman spectroscopy. *Journal of Raman Spectroscopy* **44**, 810–816 (2013).

[5] Trinh, T. T., Jansen, A. P. J. & van Santen, R. A. Mechanism of oligomerization reactions of silica. *Journal of Physical Chemistry B* **110**, 23099–23106 (2006).

[6] Hoang, V. V. Molecular dynamics simulation of amorphous SiO_2 nanoparticles. *The Journal of Physical Chemistry B* **111**, 12649–12656 (2007).

[7] Walrafen, G., Chu, Y. & Hokmabadi, M. Raman spectroscopic investigation of irreversibly compacted vitreous silica. *The Journal of Chemical Physics* **92**, 6987–7002 (1990).

[8] Hlavay, J., Jonas, K., Elek, S. & Inczedy, J. Characterization of the particle size and the crystallinity of certain minerals by ir spectrophotometry and other instrumental methods: Ii, investigations on quartz and feldspar. *Clays and Clay Minerals* **26**, 139–43 (1978).

[9] Saikia, B. J., Parthasarathy, G. & Sarmah, N. Fourier transform infrared spectroscopic estimation of crystallinity in sio2 based rocks. *Bulletin of Materials Science* **31**, 775–779 (2008).
